# No Evidence for Heritability of Male Mating Latency or Copulation Duration across Social Environments in *Drosophila melanogaster*

**DOI:** 10.1371/journal.pone.0077347

**Published:** 2013-10-14

**Authors:** Michelle L. Taylor, Jonathan P. Evans, Francisco Garcia-Gonzalez

**Affiliations:** 1 Centre for Ecology and Conservation, University of Exeter, Penryn, United Kingdom; 2 Centre for Evolutionary Biology, School of Animal Biology, University of Western Australia, Crawley, Australia; 3 Doñana Biological Station, Spanish Research Council CSIC, C/ Americo Vespucio s/n, Isla de la Cartuja, Seville, Spain; University of Massachusetts, United States of America

## Abstract

A key assumption underpinning major models of sexual selection is the expectation that male sexual attractiveness is heritable. Surprisingly, however, empirical tests of this assumption are relatively scarce. Here we use a paternal full-sib/half-sib breeding design to examine genetic and environmental variation in male mating latency (a proxy for sexual attractiveness) and copulation duration in a natural population of *Drosophila melanogaster*. As our experimental design also involved the manipulation of the social environment within each full-sibling family, we were able to further test for the presence of genotype-by-environment interactions (GEIs) in these traits, which have the potential to compromise mate choice for genetic benefits. Our experimental manipulation of the social environment revealed plastic expression of both traits; males exposed to a rival male during the sensitive period of adult sexual maturation exhibited shorter mating latencies and longer copulation durations than those who matured in isolation. However, we found no evidence for GEIs, and no significant additive genetic variation underlying these traits in either environment. These results undermine the notion that the evolution of female choice rests on covariance between female preference and male displays, an expectation that underpins indirect benefit models such as the good genes and sexy sons hypotheses. However, our results may also indicate depletion of genetic variance in these traits in the natural population studied, thus supporting the expectation that traits closely aligned with reproductive fitness can exhibit low levels of additive genetic variance.

## Introduction

Sexual selection is often thought to favour the evolution of elaborate traits that provide honest signals of (usually male) genetic quality [1,2]. According to this idea, secondary sexual traits are expected to convey reliable information about genetic quality, thus generating a correlation between signal attractiveness and the genetic benefits of choice (production of offspring of high genetic quality) which underpin good genes explanations for the evolution of female preferences [1-3]. A correlation between attractiveness and female preferences is also expected, regardless of the relationship between attractiveness and genetic quality, as long as attractive males father attractive sons; choosy females can therefore obtain genetic benefits in the form of sexy sons [4-7]. Furthermore, if attractiveness is understood as the ability of males to manipulate females into mating, models based on sexual conflict also predict that attractive males should father attractive sons [8]. Thus, a critical assumption of good genes, sexy sons, and sexual conflict models is that attractiveness should be heritable. Accordingly, sexual selection research has focused on estimating the heritability of male traits upon which female choice is based, and there is now ample support for the notion that these traits can harbour significant levels of additive genetic variation [1,9-12]. However, determining whether attractiveness itself (e.g. the probability that a male is chosen as a mate) is heritable has received far less empirical attention [13]. Ultimately we largely lack data to support this key assumption underlying models of sexual selection. 

An important complication when assessing the genetic basis of sexual signals is that their expression can depend on the social and/or physical environment in which they are expressed [14-16], such that the reliability of sexual signals in different mating contexts may be compromised [17,18]. Thus, a given genotype may express different phenotypes when expressed in different environments. Theory [19] and evidence [20-22] suggest that such genotype-by-environment interactions (GEIs) have the potential to disrupt the reliability of sexual signals in heterogeneous environments, thus potentially compromising mate choice for genetic benefits [17,19].

There is compelling evidence for the implication of indirect selection on female choice in *Drosophila* [13,23-28], and in several *Drosophila* species male sexual attractiveness – measured from mating latency – has been shown to be heritable [13,24,29]. However, these studies were conducted under homogeneous environmental conditions and therefore the influence of environmental variation on patterns of genetic variance in male mating latency is presently unknown. Yet phenotypic studies that have manipulated social environments have revealed considerable plasticity in courtship, copulation duration, and ejaculate size according to the presence/absence of rival males, which effectively manipulates the likelihood of both pre- and post- copulatory competition (i.e. mating success and sperm competition) [30-32]. A general pattern found across taxa is that males that encounter rival males either during or prior to mating respond by decreasing the time spent in courtship, increasing the length of time engaged in copulation, and increasing ejaculate size [33]. Nevertheless, it is currently unknown whether there is genetic variation underlying the plasticity of such traits in response to rival males, and thus the extent of GEIs arising from heterogeneity in social environments is presently unresolved.

In this study we determine the level of genetic variation in mating latency and copulation duration in *D. melanogaster* under varying social conditions. Specifically, we used a paternal full-sib/half-sib breeding design to generate pairs of full-sibling males that were exposed to one of two social environments; males were kept in isolation or in the company of another male during the period of early adulthood in which they reach sexual maturity. Our ensuing quantitative genetic analyses enabled us to test for genetic and environmental sources of variance underlying the expression of these traits, and for the presence of GEIs by assessing the interaction between social context and sire genotype. 

## Methods

### Ethics statement

There are no ethical issues for this non-invasive study of an invertebrate species. This species is not an endangered or protected species. Adult flies were collected on private land with the permission of Howard Park and Houghton’s wineries and then returned to the laboratory.

### Flies

Adult *Drosophila melanogaster* were collected in the Swan Valley region of Western Australia in May 2009 and maintained in the laboratory in mass populations of over 500 individuals with overlapping generations until the start of our experiment in January 2010. Populations were fed on a *Drosophila* medium containing water, oats, sugar, agar and baker’s yeast, with a sprinkling of dry yeast. Populations were kept on a 12:12hr light: dark cycle at 26°C. To generate the paternal full-sib/half-sib families for the experiment, we collected 100 virgin sires from the stock population and mated each of them to three virgin females at two-day intervals. Virgin flies were collected within 12 hours of eclosing from pupal cases and kept in separate vials for each sex for three days before the first mating. Each dam was removed from her respective laying vial three days after she had mated. From each full-sib family we collected four sons within 12 hours of eclosion and split them into two social environment treatments. These males (hereafter ‘focal males’) were either housed alone, or with an unrelated sexually mature (5-7 days old) ‘rival’ male from the stock population. All focal males were maintained in their respective social treatments for seven days during the sensitive period of adult sexual maturation. We then removed the rival male and transferred each focal male to a fresh vial with a 3-4 day old sexually mature virgin female from the stock population. We presented focal males individually to a single female to avoid potentially confounding effects of direct male-male competition on the traits measured (see below) [32]. Mating trials took place over four hours in the morning from first light, and four hours in the evening prior to dark. Siblings from both treatments were tested during the same time period to reduce variation due to time of day. All pairs of focal males were then observed continuously until copulation occurred, up to a maximum of 3 hours. A minimum of 80% of males mated within this time. For each successful copulation, we recorded the mating latency (time taken to initiate copulation) and copulation duration. Within the context of our experiment, we are interested only in the effect of perceived competition from rival males during the sexual maturation phase, rather than the effects of specific individuals on focal male mating success per se (sensu ‘interacting phenotypes’ approach [34]) and so we did not measure these behaviours in rival males. 

Our final analysis comprised data from 91 sires, 194 dams and 663 sons (mean 2.1 dams per sire; mean 3.4 sons per dam). The effects of social environment on genetic variation in male mating behaviour were examined with general linear mixed-effects models using Type III sums of squares in STATISTICA 8.0 [35]. Sire, and dam nested within sire, were included as random factors, and social environment as a fixed factor. Interactions involving random terms were also coded as random effects. We used the interaction between social environment and dam (nested within sire) as the error term for testing the significance of the sire-by-environment (i.e. GEI) interaction [36] and followed Satterthwaite’s method of denominator synthesis to account for unequal sample sizes of offspring [37]. Copulation duration was normally distributed but mating latency deviated from normality and was log- transformed prior to evaluating the sire-by-social environment interactions. We removed data for two sons as they alone generated a significant sire-by-social environment interaction for copulation duration. When we re-ran the analysis with these two males excluded from the dataset, the interaction term was no longer significant (see Results). 

To estimate genetic parameters (including heritabilities) of the behavioural traits we conducted separate analyses within each social environment treatment group. For hypothesis testing, we used a mixed-model nested ANOVA with Type III sums of squares. Variance component estimation and subsequent analyses of genetic variation were conducted using restricted maximum-likelihood (REML) in a mixed-model fitted with the lmer function (package lme4 [38]; in R 3.0.0 [39]. We used untransformed variables to analyse genetic variation [40]. We calculated narrow-sense heritabilities due to sires, heritabilities due to dams, and the genotypic (mean) estimate of heritability [41]. We calculated evolvability measures (I_A_ and the coefficient of additive genetic variation, CV_A_) [40,42,43] and coefficients of phenotypic and residual variation (CV_P_ and CV_R,_ respectively) following methods described in [40], where CV_A_ = √V_A_ / Ẋ. Finally, we calculated standard errors for heritabilities, variance components and evolvability estimates using the jack-knife procedure [41,44]. 

## Results and Discussion

### GEIs and sexual selection

**Figure 1:**
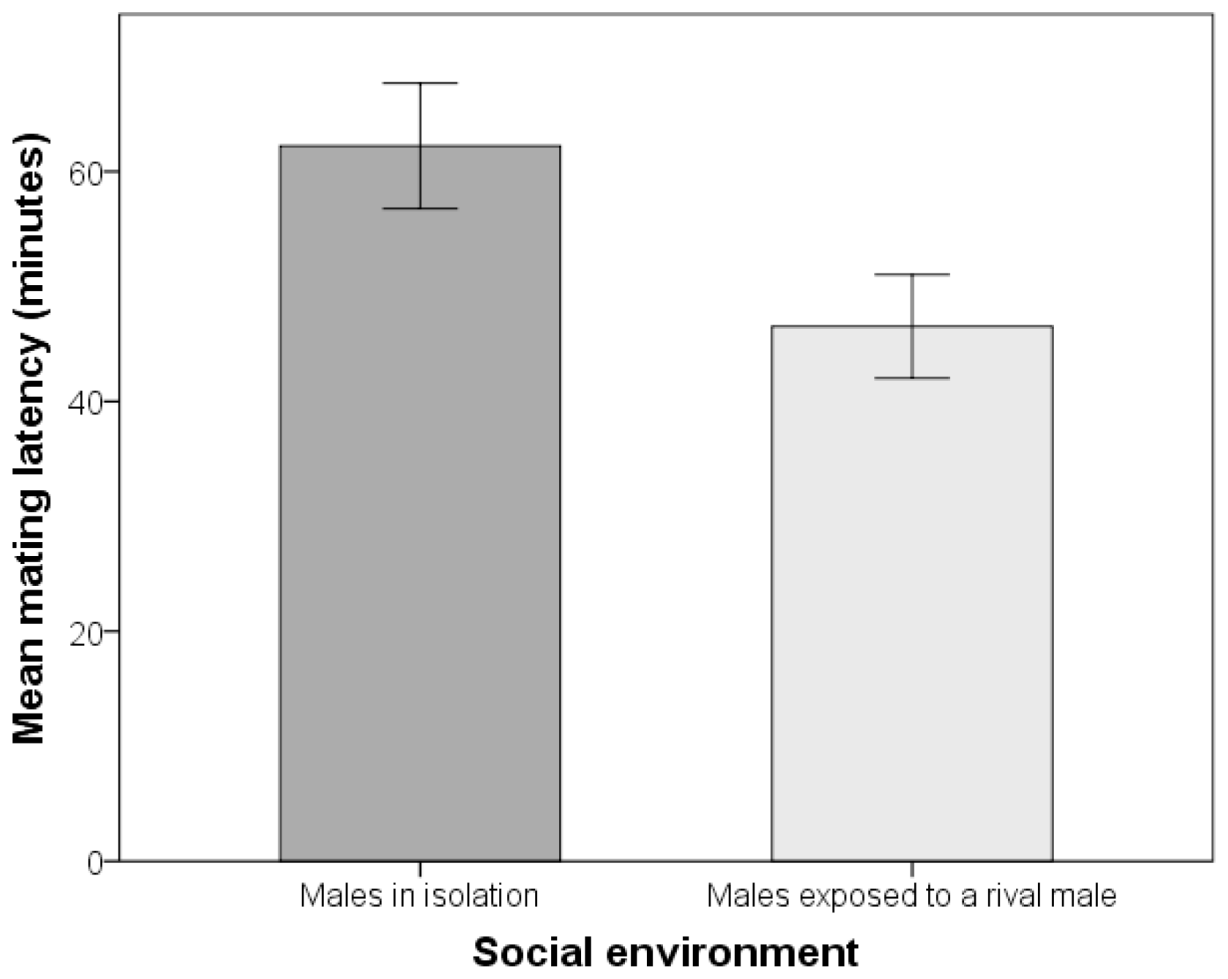
Males decrease their mating latency after being exposed to a rival male. Mean ± SE of mating latency of males in two different social environments.

**Figure 2:**
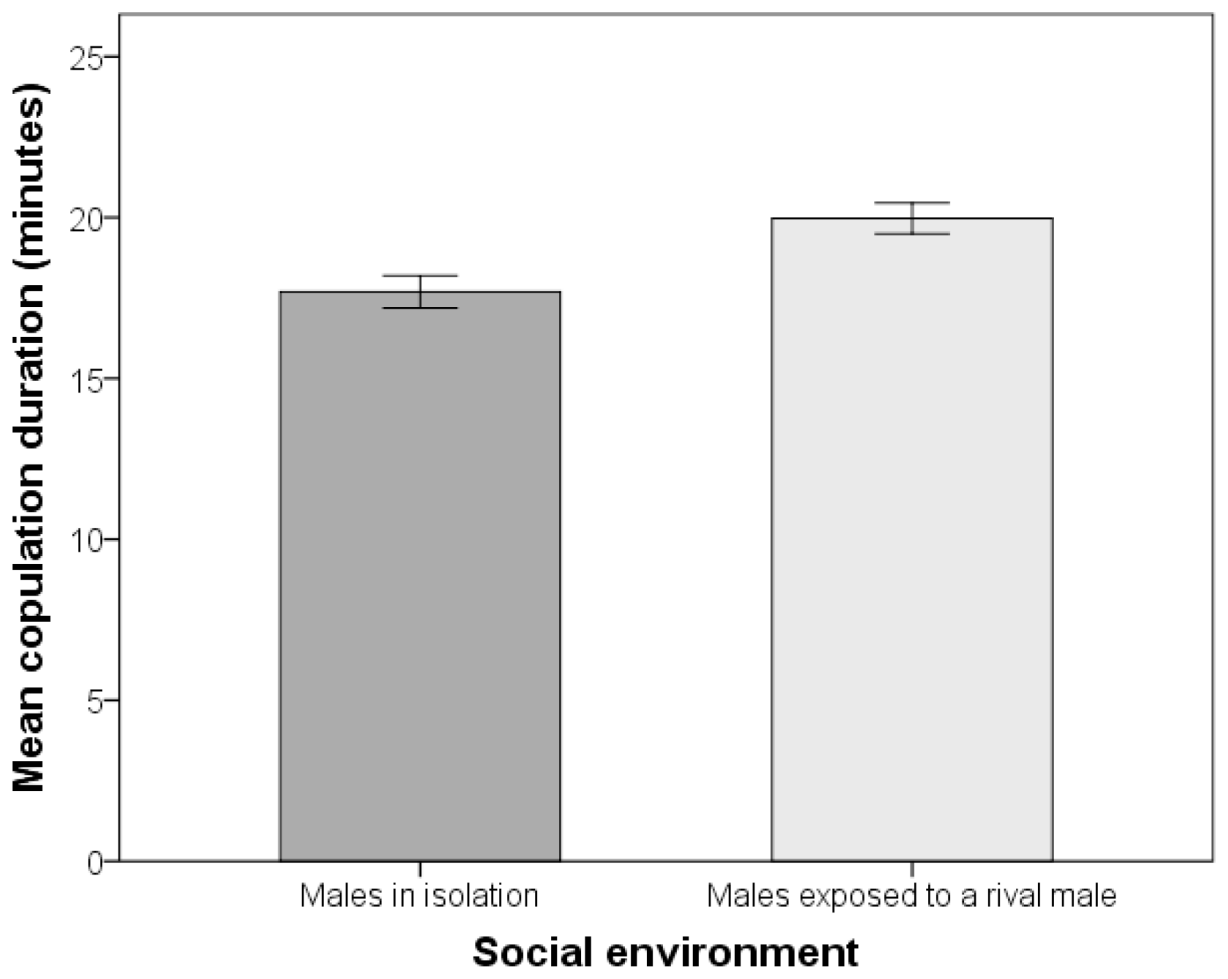
Males increase their copulation duration after being exposed to a rival male. Mean ± SE of copulation duration of males in two different social environments.

Males exposed to a rival male during sexual maturity exhibited significantly shorter mating latencies than those who matured in isolation (mean ± SE mating latencies: exposed to a rival male 46.53 ± 2.25 min., N = 341; in isolation = 62.45 ±2.74 min., N = 322; see Table 1 for supporting statistics and Figure 1). Furthermore, males exposed to a rival male had significantly longer copulation durations than males who matured in isolation (mean ± SE copulation duration: exposed to a rival male 19.97 ± 0.24 min., N = 341; in isolation = 17.69 ± 0.25 min., N = 322; Table 1 & Figure 2). Thus, we found evidence for plastic expression of these traits according to the social environment. Despite these treatment effects, we detected no significant sire effects, or additive genetic variation underlying any of the traits examined (discussed below). By contrast, we found significant variation attributable to dams for copulation duration (Table 1), but as this effect likely encompasses maternal and common environmental effects we do not interpret this finding further. There was no evidence for GEIs for either mating latency or copulation duration (see sire-by-social environment interaction; see Table 1).

**Table 1:** Mixed-model nested univariate ANOVAs for male mating latency and copulation duration.

**Trait**	**Source of variance**	**d.f.**	**MS**	**d.f. (denominator)**	**F**	**p**
*Mating*	Sire	90	0.195	38.78	1.049	.445
*latency*	Dam (Sire)	103	0.209	96.15	1.31	.091
	Social environment	1	3.214	101.19	23.23	**.000**
	Sire x Social environment	88	0.137	95.65	0.86	.759
	Dam (Sire) x Social environment	95	0.159	285	1.108	.261
	Error	285	0.144			
*Copulation*	Sire	90	33.1	70.21	1.31	.119
*duration*	Dam (Sire)	103	23.6	96.89	1.92	**.000**
	Social environment	1	850.7	100.52	60.93	**.000**
	Sire x Social environment	88	13.9	96.07	1.13	.274
	Dam (Sire) x Social environment	95	12.3	285	0.67	.987
	Error	285	18.2			

GxEs are tested via the Sire x Social environment interaction (where Dam (Sire) x Social environment term provides the error degrees of freedom). Results significant at alpha <.001 are in bold. Observed power for all tests >.84 (mating latency) and >.93 (copulation duration) (alpha = 0.05).

We found a significant effect of the social environment on both mating latency and copulation duration. Males that were exposed to a rival male during the period between eclosion and sexual maturity were more attractive to females and significantly increased their copulation duration compared to those reared in isolation. This effect was evident despite all males being tested individually in a single male mating arena with a random virgin female. The phenomenon of increased copulation duration in response to a rival male in the rearing or mating arena has been reported across many insect taxa and has been most commonly interpreted as a behavioural response to increased risk of sperm competition [32,33,45,46]. In a recent study, Lize et al. [47] compared mating latency and copulation duration under ‘single’ or ‘competitor’ mating conditions in four species of *Drosophila*. They reported that increased copulation duration in response to a competitor was a consistent response across the four species studied, but ruled out sperm competition as the selective factor underlying this response as two of the four species had extremely low levels of remating. 

One way in which males might decrease their latency to mating is to increase the effort and time spent engaged in direct courtship behaviour towards females. Previous work in *D. melanogaster* and its sibling species, *D. simulans*, has shown that males spend considerable amounts of time engaged in courtship, even to the detriment of female survival [48-50]. Another possibility is that males decreased mating latency by increasing their production of cuticular hydrocarbons [51-54]. Consistent with this idea, in Australian field crickets, *Teleogryllus oceanicus*, subdominant males have been shown to compensate for their lower status by up-regulating their investment in short-range cuticular hydrocarbons, which directly increases their mating success [55,56].

### Genetic variation in mating latency and copulation duration

We partitioned total phenotypic variance into its genetic and environmental components and estimated the heritability and evolvability of mating latency and copulation duration. We found no significant additive genetic variation underlying mating latency or copulation duration in either treatment (Tables 2 and 3). The sire and dam estimates of heritability for the two traits analysed were not significantly different from each other, and genotypic estimates were generally moderate in magnitude although not significantly different from zero. These results may seem surprising given that previous studies have found significant levels of genetic variation for these traits in this and in other species of *Drosophila* [13,29]. Nevertheless, Hoffman [29] points out that in *Drosophila*, mating speed and courtship behaviour can exhibit either low or undetectable heritabilities due to “single event testing”, where the traits in focal individuals are measured just once rather than repeatedly. However, in their meta-analysis of repeatability of animal behaviours, Bell et al. [57] report no relationship between repeatability and the number of times behaviour such as courtship was measured. 

**Table 2:** Phenotypic and genetic variation in mating latency (minutes) in males exposed to different social environments.

**Social environment**	**Mean (SE)**	**V_A_ (SE)**	**V_P_ (SE)**	**h^2^_sire_ (SE)**	**h^2^_dam_ (SE)**	**h^2^_genotypic_ (SE)**	**I_A_ (SE)**	**CV_A_ (SE)**	**CV_P_ (SE)**	**CV_R_ (SE)**
*Isolation*	62.45	2.81x10^-11^	2414.21	1.16x10^-14^	0.63	0.31	7.21x10^-15^	8.49x10^-8^	0.78	0.78
	(2.74)	(3.72 x10^-7^)	(236.92)	(1.53 x10^-10^)	(0.38)	(0.19)	(9.51 x10^-11^)	(9.65 x10^-6^)	(0.03)	(0.03)
*With rival male*	46.53	75.93	1788.42	0.04	0.58	0.31	0.035	0.18	0.90	0.87
	(2.25)	(273.30)	(234.35)	(0.16)	(0.57)	(0.29)	(0.126)	(0.45)	(0.04)	(0.08)

Coefficients of additive, phenotypic and residual variation are calculated without a 100 multiplier. Standard errors (SE) of genetic statistics were obtained by jackknifing across sires. Sample sizes for the “*isolation*” environment: n sires = 90, n dams = 187, n sons = 322; sample sizes for the “*with rival male*” environment: n sires = 90, n dams = 191, n sons = 341. Significance values are not shown as all p > 0.05.

**Table 3:** Phenotypic and genetic variation in copulation duration (minutes) in males exposed to different social environments.

**Social environment**	**Mean (SE)**	**V_A_ (SE)**	**V_P_ (SE)**	**h^2^_sire_ (SE)**	**h^2^_dam_ (SE)**	**h^2^_genotypic_ (SE)**	**I_A_ (SE)**	**CV_A_ (SE)**	**CV_P_ (SE)**	**CV_R_ (SE)**
*Isolation*	17.69	3.91	20.30	0.19	0.38	0.29	0.012	0.11	0.25	0.23
	(0.25)	(5.14)	(1.99)	(0.27)	(0.46)	(0.16)	(0.017)	(0.07)	(0.01)	(0.05)
*With rival male*	19.97	2.41x10^-9^	19.58	1.23x10^-10^	0.00	6.17x10^-11^	6.06x10^-12^	2.46x10^-6^	0.22	0.22
	(0.24)	(1.58 x10^-8^)	(2.01)	(8.15 x10^-10^)	(0.82)	(0.41)	(3.96 x10^-11^)	(7.15 x10^-6^)	(0.01)	(0.01)

Coefficients of additive, phenotypic and residual variation are calculated without a 100 multiplier. Standard errors (SE) of genetic statistics were obtained by jackknifing across sires. Sample sizes for the “*isolation*” environment: n sires = 90, n dams = 187, n sons = 322; sample sizes for the “*with rival male*” environment: n sires = 90, n dams = 191, n sons = 341. Significance values are not shown as all p > 0.05.

The problem of a potential underestimation of heritabilities may be further compounded when we consider that in order to measure focal male mating latency and copulation duration we used a random sample of females with which to measure these traits. Thus, any genetic or phenotypic variance in female preferences or female condition as well as sampling error surrounding the choice of females may inflate estimates of phenotypic variance in the traits investigated in the focal males, hence leading to an underestimation of heritability (see [58,59] for a full exposition of a similar problem in the case of estimating the heritability of sperm competitiveness). Whether this ‘noise’ is strong enough to mask the detection of significant heritability in mating latency and copulation duration in the population studied warrants further investigation, but we found consistently low levels of additive genetic variation, including mean-standardized measures of evolvability, which unlike heritability measures are independent of phenotypic variance. We also note that the sample size in our study (around 90 sire families) is similar to, or even larger than, those employed in other studies using the full-sib/half-sib design that either reported significant additive genetic variance for these traits [13,24] or were calculated as sufficient to detect significant heritability of 0.2 [60]. Finally, as our study characterises patterns of genetic variance in a natural population of *D. melanogaster*, our ensuing estimates of heritability and coefficients of additive genetic variance may reflect both high environmental variance and low additive genetic variance in natural populations compared to their lab-adapted counterparts. Conner et al. [61], for example, reported that in wild radish plants, estimates of heritability and additive genetic variance in floral traits were significantly lower in field samples compared to those coming from the laboratory. A possibility in our case is that genetic variance in male mating behaviour may have been eroded by selection in the natural population, but future studies are needed to further investigate this possibility.

## References

[1] Andersson M (1994) Sexual selection. NJ: Princeton University Press.

[2] Andersson M, Simmons LW (2006) Sexual selection and mate choice. Trends Ecol Evol 21: 296-302. doi:10.1016/j.tree.2006.03.015. PubMed: 16769428. 16769428

[3] Kokko H, Brooks R, Jennions MD, Morley J (2003) The evolution of mate choice and mating biases. Proc R Soc Lond B 270: 653-664. doi:10.1098/rspb.2002.2235. PubMed: 12769467. PMC1691281 12769467

[4] Fisher RA (1930) The Genetical Theory of Natural Selection. Oxford: Clarendon Press.

[5] Kirkpatrick M (1982) Sexual selection and the evolution of female choice. Evolution 36: 1-12. doi:10.2307/2407961. 28581098

[6] Kokko H, Brooks R, McNamara JM, Houston AI (2002) The sexual selection continuum. Proc R Soc Lond B 269: 1331-1340. doi:10.1098/rspb.2002.2020. PubMed: 12079655. PMC1691039 12079655

[7] Kokko H, Jennions MD, Brooks R (2006) Unifying and testing models of sexual selection. Annu Rev Ecol Evol Syst 37: 43-66. doi:10.1146/annurev.ecolsys.37.091305.110259.

[8] Arnqvist G, Rowe L (2005) Sexual conflict. Princeton: Princeton University Press.

[9] Norris K (1993) Heritable variation in a plumage indicator of viability in male great tits *Parus* *major* . Nature 362: 537-539. doi:10.1038/362537a0.

[10] Simmons LW (1987) Heritability of a male character chosen by females of the field cricket, *Gryllus* *bimaculatus* . Behav Ecol Sociobiol 21: 129-133. doi:10.1007/BF02395441.

[11] Houde AE (1992) Sex-linked heritability of a sexually selected character in a natural population of *Poecilia* *reticulata* (Pisces: Poecilidae) (guppies). Heredity 69: 229-235. doi:10.1038/hdy.1992.120.

[12] Hoffmann A (1991) Heritable variation for territorial success in field-collected *Drosophila* *melanogaster* . Am Nat 138: 668-679. doi:10.1086/285241.

[13] Taylor ML, Wedell N, Hosken DJ (2007) The heritability of male attractiveness. Curr Biol 17: R959-R960. doi:10.1016/j.cub.2007.09.054. PubMed: 18029248. 18029248

[14] Bussière LF, Hunt J, Stöting KN, Jennions MD, Brooks R (2008) Mate choice for genetic quality when environments vary: suggestions for empirical progress. Genetica 134: 69-78. doi:10.1007/s10709-007-9220-z. PubMed: 17987390. 17987390

[15] Higginson AD, Reader T (2009) Environmental heterogeneity, genotype-by-environment interactions and the reliability of sexual traits as indicators of mate quality. Proc R Soc Lond B 276: 1153-1159. doi:10.1098/rspb.2008.1592. PubMed: 19129106. PMC2679080 19129106

[16] Greenfield MD, Rodriguez RL (2004) Genotype-environment interaction and the reliability of mating signals. Anim Behav 68: 1461-1468. doi:10.1016/j.anbehav.2004.01.014.

[17] Ingleby FC, Hunt J, Hosken DJ (2010) The role of genotype-by-environment interactions in sexual selection. J Evol Biol 23: 2031-2045. doi:10.1111/j.1420-9101.2010.02080.x. PubMed: 20722891. 20722891

[18] Mills SC, Alatalo RV, Koskela E, Mappes J, Mappes T et al. (2007) Signal reliability compromised by genotype-by-environment interaction and potential mechanisms for its preservation. Evolution 61: 1748–1757. doi:10.1111/j.1558-5646.2007.00145.x. PubMed: 17598753. 17598753

[19] Kokko H, Heubel K (2008) Condition-dependence, genotype-by-environment interactions and the lek paradox. Genetica 132: 209-216. PubMed: 17619173. 17619173 10.1007/s10709-007-9166-1

[20] Danielson-François AM, Kelly JK, Greenfield MD (2006) Genotype x environment interaction for male attractiveness in an acoustic moth: evidence for plasticity and canalization. J Evol Biol 19: 532–542. doi:10.1111/j.1420-9101.2005.01006.x. PubMed: 16599929. 16599929

[21] David P, Bjorksten T, Fowler K, Pomiankowski A (2000) Condition-dependent signalling of genetic variation in stalk-eyed flies. Nature 406: 186-188. doi:10.1038/35018079. PubMed: 10910358. 10910358

[22] Rodríguez RL, Greenfield MD (2003) Genetic variance and phenotypic plasticity in a component of female mate choice in an ultrasonic moth. Evolution 57: 1304-1313. doi:10.1554/02-446. PubMed: 12894938. 12894938

[23] Hine E, Lachish S, Higgie M, Blows MW (2002) Positive genetic correlation between female preference and offspring fitness. Proc R Soc Lond B 269: 2215-2219. doi:10.1098/rspb.2002.2149. PubMed: 12427314. PMC1691147 12427314

[24] Hosken DJ, Taylor ML, Hoyle K, Higgins S, Wedell N (2008) Attractive males have greater success in sperm competition. Curr Biol 18: R553-R554. doi:10.1016/j.cub.2008.04.028. PubMed: 18606122. 18606122

[25] Byrne PG, Rice WR (2005) Remating in *Drosophila* *melanogaster*: an examination of the trading-up and intrinsic male-quality hypotheses. J Evol Biol 18: 1324-1331. doi:10.1111/j.1420-9101.2005.00918.x. PubMed: 16135127. 16135127

[26] Rundle HD, Odeen A, Mooers AØ (2007) An experimental test for indirect benefits in *Drosophila* *melanogaster* . BMC Evol Biol 7: 36. doi:10.1186/1471-2148-7-36. PubMed: 17349042. 17349042 PMC1828163

[27] Priest NK, Galloway LF, Roach DA (2008) Mating frequency and inclusive fitness in *Drosophila* *melanogaster* . Am Nat 171: 10-21. doi:10.1086/523944. PubMed: 18171147. 18171147

[28] Stewart AD, Hannes AM, Mirzatuny A, Rice WR (2008) Sexual conflict is not counterbalanced by good genes in the laboratory *Drosophila* *melanogaster* model system. J Evol Biol 21: 1808-1813. doi:10.1111/j.1420-9101.2008.01593.x. PubMed: 18681915. 18681915

[29] Hoffmann AA (1999) Is the heritability for courtship and mating speed in *Drosophila* (fruit fly) low? Heredity 82: 158-162. doi:10.1038/sj.hdy.6884640. PubMed: 10098264. 10098264

[30] Bretman A, Fricke C, Chapman T (2009) Plastic responses of male *Drosophila* *melanogaster* to the level of sperm competition increase male reproductive fitness. Proc R Soc Lond B 276: 1705–1711. doi:10.1098/rspb.2008.1878. PubMed: 19324834. PMC2660996 19324834

[31] Wigby S, Sirot LK, Linklater JR, Buehner N, Calboli FCF et al. (2009) Seminal fluid protein allocation and male reproductive success. Curr Biol 19: 751-757. doi:10.1016/j.cub.2009.03.036. PubMed: 19361995. 19361995 PMC2737339

[32] Bretman A, Gage MJG, Chapman T (2011) Quick-change artists: male plastic behavioural responses to rivals. TREE 26: 467-473. PubMed: 21680050. 21680050 10.1016/j.tree.2011.05.002

[33] Kelly CD, Jennions MD (2011) Sexual selection and sperm quantity: meta-analyses of strategic ejaculation. Biol Rev 86: 863-884. doi:10.1111/j.1469-185X.2011.00175.x. PubMed: 21414127. 21414127

[34] Moore AJ, Brodie ED, Wolf JB (1997) Interacting phenotypes and the evolutionary process: I. Direct and indirect genetic effects of social interactions. Evolution 51: 1352-1362. doi:10.2307/2411187. 28568644

[35] StatSoft Inc. (2007) tatistica (data analysis software system), version 8.0. Available: www.statsoft.com. Accessed 2013 September 20.

[36] Watson NL, Simmons LW (2012) Unravelling the effects of differential maternal allocation and male genetic quality on offspring viability in the dung beetle, *Onthophagus* *sagittarius* . Evol Ecol 26: 139-147. doi:10.1007/s10682-011-9484-8.

[37] Lynch M, Walsh B (1998) Genetics and Analysis of Quantitative Traits. Sunderland: Sinauer Associates, Inc.

[38] Bates D, Maechler M, Bolker B (2013) me4: Linear mixed-effects models using S4 classes. R package version 0.999999-2.

[39] Core R Team (2013) R: A language and environment for statistical computing. Vienna, Austria: R Foundation for Statistical Computing . Available: http://www.R-project.org/ . Accessed 2013 September 20

[40] Garcia-Gonzalez F, Simmons LW, Tomkins JL, Kotiaho JS, Evans JP (2012) Comparing evolvabilities: common errors surrounding the calculation and use of coefficients of additive genetic variation. Evolution 66: 2341-2349. doi:10.1111/j.1558-5646.2011.01565.x. PubMed: 22834736. 22834736

[41] Roff DA (2008) Comparing sire and dam estimates of heritability: jackknife and likelihood approaches. Heredity 100: 32-38. doi:10.1038/sj.hdy.6801048. PubMed: 17786161. 17786161

[42] Houle D (1992) Comparing evolvability and variability of quantitative traits. Genetics 130: 195-204. PubMed: 1732160. 1732160 10.1093/genetics/130.1.195 PMC1204793

[43] Hansen T, Pélabon C, Houle D (2011) Heritability is not evolvability. Evol Biol 38: 258-277. doi:10.1007/s11692-011-9127-6.

[44] Roff DA (2006) Introduction to Computer-Intensive Methods of Data Analysis in Biology. Cambridge: Cambridge University Press.

[45] Weir LK, Grant JWA, Hutchings JA (2011) The influence of operational sex ratio on the intensity of competition for mates. Am Nat 177: 167–176. doi:10.1086/659946. PubMed: 21460553. 21460553

[46] Garcia-Gonzalez F, Gomendio M (2004) Adjustment of copula duration and ejaculate size according to the risk of sperm competition in the golden egg bug (*Phyllomorpha* *laciniata*). Behav Ecol 15: 23-30. doi:10.1093/beheco/arg095.

[47] Lizé A, Doff RJ, Smaller EA, Lewis Z, Hurst GDD (2012) Perception of male-male competition influences *Drosophila* copulation behaviour even in species where females rarely remate. Biol Lett 8: 35-38. doi:10.1098/rsbl.2011.0544. PubMed: 21752815. 21752815 PMC3259955

[48] Partridge L, Fowler K (1990) Non-mating costs of exposure to males in female *Drosophila* *melanogaster* . J Insect Physiol 36: 419-425. doi:10.1016/0022-1910(90)90059-O.

[49] Taylor ML, Wigmore C, Hodgson DJ, Wedell N, Hosken DJ (2008) Multiple mating increases female fitness in *Drosophila* *simulans* . Anim Behav 76: 963-970. doi:10.1016/j.anbehav.2008.05.015.

[50] Taylor ML, Wedell N, Hosken DJ (2008) Sexual selection and female fitness in *Drosophila* *simulans* . Behav Ecol Sociobiol 62: 721-728. doi:10.1007/s00265-007-0497-9.

[51] Kent C, Azanchi R, Smith B, Formosa A, Levine JD (2008) Social context influences chemical communication in *D.* *melanogaster* males. Curr Biol 18: 1384-1389. doi:10.1016/j.cub.2008.07.088. PubMed: 18789689. 18789689

[52] Petfield D, Chenoweth SF, Rundle HD, Blows MW, Avise JC (2005) Genetic variance in female condition predicts indirect genetic variance in male sexual display traits. Proc Natl Acad Sci USA 102: 6045-6050. doi:10.1073/pnas.0409378102. PubMed: 15840726. 15840726 PMC1087918

[53] Svetec N, Ferveur J-F (2005) Social experience and pheromonal perception can change male-male interactions in *Drosophila* *melanogaster* . J Exp Biol 208: 891-898. doi:10.1242/jeb.01454. PubMed: 15755887. 15755887

[54] Svetec N, Cobb M, Ferveur J-F (2005) Chemical stimuli induce courtship dominance in *Drosophila* . Curr Biol 15: R790-R792. doi:10.1016/j.cub.2005.09.034. PubMed: 16213806. 16213806

[55] Thomas ML, Simmons LW (2009) Male dominance influences pheromone expression, ejaculate quality, and fertilization success in the Australian field cricket, *Teleogryllus* *oceanicus* . Behav Ecol 20: 1118-1124. doi:10.1093/beheco/arp105.

[56] Thomas ML, Gray B, Simmons LW (2011) Male crickets alter the relative expression of cuticular hydrocarbons when exposed to different acoustic environments. Anim Behav 82: 49-53. doi:10.1016/j.anbehav.2011.03.023.

[57] Bell AM, Hankison SJ, Laskowski KL (2009) The repeatability of behaviour: a meta-analysis. Anim Behav 77: 771-783. doi:10.1016/j.anbehav.2008.12.022. 24707058 PMC3972767

[58] García-González F (2008) The relative nature of fertilization success: implications for the study of post-copulatory sexual selection. BMC Evol Biol 8: 140. doi:10.1186/1471-2148-8-140. PubMed: 18474087. 18474087 PMC2408597

[59] Garcia-Gonzalez F, Evans JP (2011) Fertilization success and the estimation of genetic variance in sperm competitiveness. Evolution 65: 746-756. doi:10.1111/j.1558-5646.2010.01127.x. PubMed: 20880262. 20880262

[60] Boake CRB, Konigsberg L (1998) Inheritance of male courtship behaviour, aggressive success, and body size in *Drosophila* *silvestris* . Evolution 52: 1487-1492. doi:10.2307/2411318. 28565381

[61] Conner JK, Franks R, Stewart C (2003) Expression of additive genetic variances and covariances for wild radish floral traits: Comparison between field and greenhouse environments. Evolution 57: 487-495. doi:10.1111/j.0014-3820.2003.tb01540.x. PubMed: 12703938. 12703938

